# Supplementary material for: Investigating SnOx/Graphene Oxide heterostructure for methane sensing and its application as a tunable light absorber for optoelectronic devices
Source: PLoS One. 2025 Jul 3;20(7):e0326657. doi: 10.1371/journal.pone.0326657 (PMC12225864; doi:10.1371/journal.pone.0326657)
Supplement: Attachment — Submitted filename: response to reviewers_plos_one_revised.pdf [file pone.0326657.s003.pdf]

Original Manuscript ID: PONE-D-25-15120

Original Article Title: "Investigating SnOx/Graphene Oxide Heterostructure for Methane Sensing and Its Application as a Tunable Light Absorber for Optoelectronic Devices"

To: PLOS one , Editor

Re: Response to reviewers

Dear Editor,

Thank you for allowing a resubmission of our manuscript, with an opportunity to address the reviewers' comments.

We are uploading (a) our point-by-point response to the comments (below) (response to reviewers, under "Author's Response Files"), (b) an updated manuscript with yellow highlighting indicating changes (as "Highlighted PDF"), and (c) a clean updated manuscript without highlights ("Main Manuscript"*).*

Best regards,

Manoj Kumar et al.

Reviewer#1

Reviewer#1, Comment # 1: In the article, the SnO_x/GO heterojunctions are classified into four types (Type-A to Type-D), but the specific numerical range or experimental determination method of oxygen content (x) is not clarified. It is only described vaguely as "high/low". Please use computational or experimental evidence to prove the specific numerical differences in the oxygen content of the four types of heterojunctions, please mark the chemical formula of SnO_x or the range of the molar fraction of oxygen in the chart (as shown in Fig.1).

Author response: Thank you for your comments and your thorough evaluation of our manuscript.

We have performed Density Functional Theory (DFT) simulations to model SnO_x molecules with varying oxygen content (x). The non-stoichiometry of SnO_x was systematically adjusted by modifying the relative proportions of SnO and SnO_2 [18], [19].

For each compositional ratio, we calculated the corresponding value of (x), representing the oxygen mole fraction or the average number of oxygen atoms per Sn atom. In this study, we have considered four different ratios of SnO_2 and SnO to systematically vary the oxygen content in SnO_x, resulting in compositions with (x)=1.5, (x)=1.33, (x)=1.25, and (x)=1.20.

These non-stoichiometric compositions represent intermediate states between pure SnO_2 and SnO are categorized as follows: $SnO_{1.5}$ (Type A, oxygen-rich), $SnO_{1.33}$ (Type B), $SnO_{1.25}$ (Type C), and $SnO_{1.20}$ (Type D, oxygen-deficient).

The $SnO_{1.5}$ corresponds to a mixture of 1 mole of SnO and 1 mole of SnO_2, yielding an average oxygen-to-tin ratio (x)=1.5. The value of oxygen mole fraction (x) is calculated using the formula:

(x)=Number of O atoms in the composition/Number of Sn atoms in the composition

In this case, the total O atoms = 1 Oxygen atoms from SnO + 2 Oxygen atoms from SnO_2 = 3 and the total Sn atoms = 1 from SnO + 1 from SnO_2 = 2, yielding (x)=3/2=1.5.

Similarly, a composition of $SnO_{1.33}$ corresponds to a mixture of 1 mole of SnO_2 and 2 moles of SnO, yielding an average oxygen-to-tin ratio (x)=1.33. For $SnO_{1.25}$, the mixture consists of 1 mole of SnO_2 and 3 moles of SnO, resulting in (x)=1.25. Likewise, $SnO_{1.20}$ represents a composition with 1 mole of SnO_2 and 4 moles of SnO, giving an average value of (x)=1.20. Each of these compositions reflects a different ratio of SnO_2 to SnO and is used to model non-stoichiometric SnO_x phases by varying the oxygen content systematically.

Author action: We updated the manuscript by modifying/adding some sentences at 4 of the revised manuscript.

Reviewer#1, Comment # 2: The phenomenon of increased reflectivity and decreased absorption caused by methane adsorption is only attributed to "electron interaction". Please use DFT to explain what factors the interaction sources of these phenomena are related to, such as charge density, etc.

Author response: Thank you for your comments and your thorough evaluation of our manuscript.

The phenomenon of increased reflectivity and decreased photo absorption caused by methane adsorption on the surface of graphene oxide can be primarily attributed to changes in charge density, density of states (DOS), and the dielectric constant. When methane molecules adsorb onto the graphene oxide surface, they induce a redistribution of electronic charge at the interface, altering the

local charge density. This redistribution modifies the electronic environment, leading to subtle shifts in the DOS, particularly near the Fermi level, which affects the probability of electronic transitions under incident light [23]. As shown in the Density of states (DOS) plots in Figure 5, an increase in methane concentration (from Type-I to Type-IV) results in significant modifications in the electronic structure of the SnO_x/GO heterostructure. Specifically, with higher CH_4 adsorption, the density of states near the Fermi level becomes more pronounced, indicating stronger electronic interactions between methane molecules and the sensing surface. These changes can be attributed to enhanced charge transfer between methane and the SnO_x/GO system. At low concentrations, methane introduces only slight perturbations to the DOS, mostly due to weak physisorption. However, as concentration increases, stronger chemisorption occurs, leading to additional localized states within the bandgap and near the Fermi level. This results in a shift in the Fermi level results in an increased in charge redistribution and electronic coupling between adsorbed methane and surface atoms. These DOS changes directly impact the material's photoabsorption and sensing behavior. The introduction of new states modifies the allowed optical transitions, increasing the absorption of visible and UV light. These findings are consistent with reported trends in literature, where increased DOS near the Fermi level upon gas adsorption correlates with improved sensing properties [24], [25]. Furthermore, these electronic changes influence the material's complex dielectric constant—specifically, the real part (ε_1)which governs reflectivity, and the imaginary part (ε_2), which determines absorption. Methane adsorption tends to reduce ε_2 resulting in lower optical absorption, while changes in ε_1 can enhance reflectivity [26].

Author action: We updated the manuscript by modifying/adding some sentences at page 9 and 10 of the revised manuscript.

Reviewer#1, Comment # 3: The abscissa of Figure 6 is kcal/mol, and it does not mention what kind of energy it is. Adsorption energy? Binding energy? Or something else?

Author response: Thank you for your comments and your thorough evaluation of our manuscript.

We acknowledge the ambiguity and have now clarified that the energy values presented in Figure 6 (now Figure 7) represent adsorption energy ($E_{\_ads}$) in kcal/mol obtained from adsorption locator tool of Material Studio.

Author action: We updated the manuscript by modifying/adding some sentences at page 7 of the revised manuscript.

Reviewer#1, Comment # 4: The main text mentioned the use of Monte Carlo calculation, but did not mention the Settings of relevant parameters such as temperature and pressure. Please provide the detailed parameters.

Author response: Thank you for your comments and your thorough evaluation of our manuscript.

It may be mentioned here that Adsorption Locator tool that uses Monte Carlo calculation has been used to identify possible adsorption configurations of SnO_x/GO heterostructure. The Monte Carlo calculation parameters were set to standard ambient conditions, with a temperature of 298 K and a pressure of 1 atm. The simulation has been carried out using Universal Force Field with a cutoff radius of 3 Å applied for van der Waals interactions. Additionally, the number of simulation steps was set to 100,000, ensuring sufficient sampling for equilibrium and reliable results. These parameters were selected to provide a realistic assessment of gas–surface interactions [20].

Author action: We updated the manuscript by modifying/adding some sentences at page 5 and 6 of the revised manuscript.

Reviewer#1, Comment # 5: The calculation details of CASTEP are too few. The selection of pseudopotentials, whether van der Waals dispersion correction was chosen, and the sampling of K-points have not all been provided in the main text.

Author response: Thank you for your comments and your thorough evaluation of our manuscript.

We appreciate the feedback and understand the need for more clarity regarding the computational details. In response, we have provided additional information about the CASTEP calculations in the manuscript. The calculations have been performed using generalized gradient approximation (GGA) with the Perdew-Burke-Ernzerhof (PBE) functional by norm-conserving pseudopotentials of Cambridge Sequential Total Energy Package code (CASTEP) tool kit of Material Studio. Specifically, norm-conserving pseudopotentials has been used for all elements and incorporated Grimme's correction for van der Waals interactions in all calculations to account for long-range dispersion forces. Regarding k-point sampling, we set the grid to 2×2×2 for Brillouin zone integration in bulk systems, ensuring sufficient accuracy for the simulations while maintaining computational efficiency. The convergence tolerance parameters value was set to be as 830 eV as cutoff energy for the k-point with fine mesh, maximum force, maximum stress and maximum displacement were set to be 0.03 eV/A^0, 0.05 GPa and 0.001 Å, respectively. The SCF tolerance value was considered to be fine, i.e., 10^{-9} eV/atom.

Author action: We updated the manuscript by modifying/adding some sentences at page 3 of the revised manuscript.

Reviewer#1, Comment # 6: The unit cell parameters and vacuum layer of the four heterogeneous structures are not provided.

Author response: Thank you for your comments and your thorough evaluation of our manuscript.

It may be mentioned here that the dimensions of the simulation cells for each configuration of SnO_x/GO heterostructure has an in-plane unit cell of approximately 15 Å × 15 Å with a vacuum layer of 20 Å along the z-axis.

Author action: We updated the manuscript by modifying/adding some sentences in the abstract at page 3 of the revised manuscript.

Reviewer#1, Comment # 7: Please unify the use of picture descriptions in the main text and use them uniformly in accordance with the regulations of the journal, such as Fig. or Figure.

Author response: Thank you for highlighting this formatting issue.

All figure references have been standardized according to journal guidelines, using "Figure" throughout the main text.

Author action: We updated the manuscript by modifying/adding some sentences in the revised manuscript.

Reviewer#1, Comment # 8: The resolution of Figure 1 and Figure 2 is too low, please give the color scale in the figures.

Author response: Thank you for your comments and your thorough evaluation of our manuscript.

Figures 1 and 2 have been updated to high-resolution formats and now include clearly labeled atom names for better interpretability.

Author action: We updated the figures at page 5 and 6 of the revised manuscript.

Reviewer#2

Reviewer#2, Comment # 1: The synthesis method of SnOx/GO composites is not mentioned in this work. At least, referencing established preparation approaches from prior studies should be included. Additionally, the material stability under prolonged methane exposure and ambient storage remains unvalidated.

Author response: Thank you for your comments and your thorough evaluation of our manuscript.

To support the relevance of our simulation study for experimental exploration, it is important to reference established synthesis methods and stability evaluations of SnO_x/graphene oxide (GO) composites. While this study is centered on theoretical investigations, it builds upon a strong foundation of experimentally validated synthesis methods for SnO_x/GO-based heterostructures, which are well-documented in existing literature. According to literature, SnO_x/GO nanocomposites have been effectively synthesized using straightforward and cost-efficient methods, including room-temperature solution-based approaches and hydrothermal techniques [2], [11]. A single-step method employing a domestic pressure cooker has also been reported for the fabrication of reduced graphene oxide/SnO_2 (RGO/SnO_2) composites, demonstrating the feasibility of scalable synthesis [12] Furthermore, a combination of sol-gel and hydrothermal methods has been used to produce SnO_2/rGO composites with tunable dielectric properties, directly relevant to sensor applications [13].In terms of material stability, experimental evidence indicates that SnO_2/graphene-based sensors maintain excellent methane sensing performance and structural integrity over extended testing periods [14]. Regarding its stability, multiple studies have consistently demonstrated the robust and durable performance of SnO_x/GO-based materials when exposed to methane under ambient conditions. In a study, it is reported that GO-SnO_2 composites exhibits stable methane sensing responses without significant degradation, even after repeated testing and prolonged ambient storage [15]. Supporting this, an earlier study confirmed the long-term operational reliability of related nanocomposite systems, emphasizing their durability [16]. Furthermore, additional evidence proves that Pd-doped SnO_2/rGO nanocomposites maintained consistent sensing behavior over long periods at room temperature [17]. Collectively, these findings affirm the suitability of SnO_x/GO-based sensors for reliable and sustained methane detection in practical applications. Ultimately, this research aims to pave the way for the next generation of sensors and devices, offering improved performance and functionality by uncovering the core mechanisms behind the gas sensing and optoelectronic capabilities of SnO_x-GO heterostructure.

Author action: We updated the manuscript by modifying/adding some sentences at page 2 and 3 of the revised manuscript.

Reviewer#2, Comment # 2: The conclusions of this study are solely based on computational simulations, lacking essential experimental validation. For instance, the actual methane-sensing performance of SnOx/GO heterostructure has not been experimentally evaluated. We recommend supplementing experimental verification or citing relevant experimental studies on analogous systems to evaluate the reliability of the computational findings.

Author response: Thank you for your comments and your thorough evaluation of our manuscript.

We acknowledge that the conclusions presented in this study are derived entirely from computational simulations, which indeed warrant experimental validation for comprehensive reliability. While direct experimental testing of the methane-sensing performance of the SnO_x/GO heterostructure is beyond the scope of the current work, we have taken steps to bridge this gap by referencing relevant experimental studies on analogous systems. Although this work does not detail about the experimental validation but several experimental studies offer well-documented approaches. According to literature, SnO_x/GO nanocomposites have been effectively synthesized using straightforward and cost-efficient methods, including room-temperature solution-based approaches and hydrothermal techniques [2], [11]. A single-step method employing a domestic pressure cooker has also been reported for the fabrication of reduced graphene oxide/SnO_2 (RGO/SnO_2) composites, demonstrating the feasibility of scalable synthesis [12] Furthermore, a combination of sol-gel and hydrothermal methods has been used to produce SnO_2/rGO composites with tunable dielectric properties, directly relevant to sensor applications [13].

We also agree that future experimental validation is essential and plan to pursue this in subsequent studies.

Author action: We updated the manuscript by modifying/adding some sentences at page 2 and 3 of the revised manuscript.

Reviewer#2, Comment # 3: Although computational simulations validate that the SnOx/GO heterojunction enables ppm-level trace methane detection, it fails to specify the exact limit of detection (LOD) or provide experimental validation. Furthermore, a comparative analysis with mainstream methane sensors in terms of sensitivity, or LOD is notably absent.

Author response: Thank you for your insightful comment.

We appreciate the observation regarding the lack of precise specification for the limit of detection (LOD) and the absence of experimental validation in our study. While our computational simulations builds a strong foundation by exploring key properties of SnO_x/GO heterojunction and guiding material selection, we acknowledge that the exact limit of detection (LOD) for methane was not clearly established in this study. Our results indicate that the SnO_x/GO heterojunction holds promise for efficient methane detection; however, defining its precise sensing capabilities requires experimental validation. These aspects, such as determining sensitivity, LOD, and sensor performance under real-world conditions are beyond the scope of the current theoretical work and will be addressed in future studies through experimental investigations.

However, in this theoretical study , it has been observed that the lower energy state (such as -78.35 kcal/mol) typically corresponds to a more stable adsorption configuration, indicating stronger interactions between methane and the SnO_x/GO surface. This stronger interaction is likely due to enhanced adsorption strength, which can influence the methane sensing performance by improving the sensitivity of the material to trace amounts of methane [34]. Stronger adsorption generally implies that the material can detect and adsorb methane molecules more effectively, leading to better sensitivity and response. Therefore, this lower energy state could enhance the material's ability to detect even low concentrations of methane, potentially lowering the limit of detection (LOD) and improving overall sensor performance [35].

Author action: We updated the manuscript by modifying/adding some sentences at page 2 , 3 and 13 of the revised manuscript.

Reviewer#2, Comment # 4: The selectivity validation is absent, as the study exclusively examines methane response without addressing possible interference from co-existing environmental gases (e.g., CO2 and H2O). Neither experimental evaluations nor computational analyses are provided to confirm the material's specificity under practical operating conditions.

Author response: We thank the reviewer for this valuable observation.

In the revised manuscript, we have addressed the concern regarding selectivity by incorporating additional computational analysis using the Adsorption Locator tool in BIOVIA Materials Studio. Specifically, we have evaluated the adsorption energies of methane (CH_4), carbon dioxide (CO_2), and water (H_2O) on the SnO_x/graphene oxide surface.

Specifically, we have evaluated the adsorption energies of methane (CH_4), carbon dioxide (CO_2), and water (H_2O) on the SnO_x/GO surface. Our simulation results indicate that methane exhibits a significantly higher adsorption energy of approximately −78.35 kcal/mol, compared to −42.75 kcal/mol for H_2O and −39.65 kcal/mol for CO_2. These results suggest that CH_4 interacts more strongly with the sensing surface than the potential interfering gases, supporting the material's preferential affinity for methane. Therefore, under typical environmental conditions, the likelihood of interference from ambient CO_2 and H_2O is minimal. This computational validation reinforces the sensor's selectivity and supports its suitability for practical methane detection applications. Moreover, these energy distributions provide important insight into the stability and effectiveness of the sensor. The discrete energy states correspond to different adsorption sites or surface configurations of the SnO_x/GO heterojunction [31]. Specifically, a more negative adsorption energy (such as -78.35 kcal/mol) indicates stronger affinity of methane molecules to the surface of SnO_x/GO heterostructure, which is typically associated with higher sensing sensitivity and better charge transfer efficiency[32]. This enhanced interaction can influence the local electronic structure, leading to measurable changes in optical properties such as the refractive index and extinction coefficient are key indicators in optical methane sensing. The energy states observed in the system (-78.35, −42.75, and −39.65 kcal/mol) are indeed crucial for understanding the adsorption stability and charge transfer mechanisms of the SnO_x/GO heterojunction in methane sensing or photoabsorption processes. Charge transfer between methane molecules and the surface could lead to changes in the electronic properties of the material, impacting its photoabsorption and methane sensing capabilities [33].

The lower energy state (such as -78.35 kcal/mol) typically corresponds to a more stable adsorption configuration, indicating stronger interactions between methane and the SnO_x/GO surface. This stronger interaction is likely due to enhanced adsorption strength, which can influence the methane sensing performance by improving the sensitivity of the material to trace amounts of methane [34]. Stronger adsorption generally implies that the material can detect and adsorb methane molecules more effectively, leading to better sensitivity and response. Therefore, this lower energy state could enhance the material's ability to detect even low concentrations of methane, potentially lowering the limit of detection (LOD) and improving overall sensor performance [35].

Author action: We updated the manuscript by modifying/adding some sentences at page 13 and 14 of the revised manuscript.

Reviewer#2, Comment # 5: The study highlights the application value of this research for photoelectrochemical systems and device engineering, yet fails to specify practical implementation strategies for SnOx/GO heterojunctions in such applications.

Author response: Thank you for your comments and valuable suggestion.

In the revised manuscript, we have elaborated on possible integration pathways of SnO_x/GO heterojunctions in real optoelectronic devices. The SnO_x/GO heterojunctions represent a promising class of materials for photoelectrochemical applications, particularly in solar energy conversion systems such as water splitting and CO_2 reduction. This heterostructure take advantage of its high

surface area and electronic properties of graphene oxide (GO), combined with the semiconducting characteristics of tin oxide (SnO_x), making them highly efficient in converting light energy into chemical energy. The integration of these two components creates a material with excellent charge separation, enhanced conductivity, and increased photocatalytic activity, making it suitable for use as photoanodes or catalysts in photoelectrochemical cells.

While this study focuses on theoretical investigations, existing experimental research provides a solid foundation that aligns with our theoretical findings. Several studies in the literature have demonstrated practical implementation strategies for SnO_2/GO heterojunctions in photoelectrochemical (PEC) applications. These experimental works offer valuable insights into synthesis techniques, device fabrication, and performance characteristics, supporting the potential integration of SnO_2/GO heterostructures into real-world PEC systems. For instance, earlier studies have shown that hydrothermal processing is a widely used method for synthesizing SnO_2/GO nanocomposites, which significantly enhance photocatalytic and electronic performance. Also, it has been shown that SnO_2/GO composites synthesized via this method exhibited improved photocatalytic activity due to enhanced light absorption and charge separation efficiency [2] .

Additionally, the sol-gel method has been employed for precise morphological control, which is crucial for tuning the heterojunction's properties. This approach has been used to fabricate SnO_2/GO heterostructure which are capable of visible-light-driven photocatalytic degradation, highlighting their potential in environmental remediation and solar-driven chemical reactions. Similarly, chemical vapor deposition (CVD) has been successfully used to grow high-quality SnO_2 thin films on GO substrates for optoelectronic applications, demonstrating the scalability and compatibility of CVD for device fabrication [2].

In terms of device integration, a self-powered UV photodetector based on SnO_2 nanorods combined with Ag/reduced graphene oxide (rGO) has been developed, showcasing the potential of SnO_x/GO-based structures in advanced optoelectronic devices [36]. Furthermore, it has been demonstrated that rGO-SnO_2 nanocomposites exhibited strong photocatalytic degradation of methylene blue under both UV and natural sunlight, suggesting their application in waste water treatment and other environmental technologies [37].

Author action: We updated the manuscript by modifying/adding some sentences at page 14 of the revised manuscript.

Reviewer#2, Comment # 6: The manuscript contains redundant citations, with References [19], [20], and [23] all referring to the same publication. We recommend delete these duplicate references to comply with standardized citation practices.

Author response: Thank you for pointing out redundant citations.

In the revised manuscript we have carefully reviewed the reference list and removed the redundant citations. The reference numbers have been updated accordingly to ensure consistency throughout the manuscript.

Author action: We updated the reference list at page 16 of the revised manuscript.

Reviewer#2, Comment # 7: Some phrasing issues in the manuscript should be further improved before publication.

Author response: Thank you for your comments and your thorough evaluation of our manuscript.

We appreciate this constructive feedback. The entire manuscript has undergone thorough language polishing to enhance readability, grammatical correctness, and scientific clarity. We have revised

sentence structures, eliminated redundancy, and improved technical phrasing, particularly in the Abstract, Methods, and Conclusion sections.

Author action: We updated the manuscript by modifying/adding some sentences in the revised manuscript.

Reviewer#3

Reviewer#3, Comment # 1: The energy distribution analysis shows that the system stabilizes at three discrete energy states of -8.3, -8.8, and -9.4 kcal/mol. However, how do these energy states specifically influence the photoabsorption or methane sensing performance of the SnOx/GO system? For instance, does a lower energy value (such as -9.4 kcal/mol) indicate stronger adsorption stability? Do the different energy states correspond to particular surface configurations or charge transfer mechanisms?

Author response: Thank you for your insightful comments and for carefully evaluating our manuscript.

The discrete energy states initially mentioned in the manuscript (-8.3, -8.8, and -9.4 kcal/mol) were due to a typographical error. In the revised version, we have corrected these values to reflect the actual adsorption energies obtained from our computational simulations.

Specifically, we have evaluated the adsorption energies of methane (CH_4), carbon dioxide (CO_2), and water (H_2O) on the SnO_x/GO surface. Our simulation results indicate that methane exhibits a significantly higher adsorption energy of approximately −78.35 kcal/mol, compared to −42.75 kcal/mol for H_2O and −39.65 kcal/mol for CO_2. These results suggest that CH_4 interacts more strongly with the sensing surface than the potential interfering gases, supporting the material's preferential affinity for methane. Therefore, under typical environmental conditions, the likelihood of interference from ambient CO_2 and H_2O is minimal. This computational validation reinforces the sensor's selectivity and supports its suitability for practical methane detection applications. Moreover, these energy distributions provide important insight into the stability and effectiveness of the sensor. The discrete energy states correspond to different adsorption sites or surface configurations of the SnO_x/GO heterojunction [31]. Specifically, a more negative adsorption energy (such as -78.35 kcal/mol) indicates stronger affinity of methane molecules to the surface of SnO_x/GO heterostructure, which is typically associated with higher sensing sensitivity and better charge transfer efficiency[32]. This enhanced interaction can influence the local electronic structure, leading to measurable changes in optical properties such as the refractive index and extinction coefficient are key indicators in optical methane sensing. The energy states observed in the system (-78.35, −42.75, and −39.65 kcal/mol) are indeed crucial for understanding the adsorption stability and charge transfer mechanisms of the SnO_x/GO heterojunction in methane sensing or photoabsorption processes. Charge transfer between methane molecules and the surface could lead to changes in the electronic properties of the material, impacting its photoabsorption and methane sensing capabilities [33].

The lower energy state (such as -78.35 kcal/mol) typically corresponds to a more stable adsorption configuration, indicating stronger interactions between methane and the SnO_x/GO surface. This stronger interaction is likely due to enhanced adsorption strength, which can influence the methane sensing performance by improving the sensitivity of the material to trace amounts of methane [34]. Stronger adsorption generally implies that the material can detect and adsorb methane molecules more effectively, leading to better sensitivity and response. Therefore, this lower energy state could enhance the material's ability to detect even low concentrations of methane, potentially lowering the limit of detection (LOD) and improving overall sensor performance [35].

Author action: We updated the manuscript by modifying/adding some sentences in the abstract at page 1,6,7,8 and 13 of the revised manuscript.

Reviewer#3, Comment # 2: The paper emphasizes the high sensitivity of the Type-II heterostructure in methane sensing (e.g., extinction coefficient up to 8.0), but lacks experimental validation data such as response time, selectivity, and repeatability. For example, is this material affected by humidity or interference from other gases in real environments? Are there practical sensor device tests (e.g., detection limit, linear range)? Is there a gap between theoretical simulation results and actual sensing performance?

Author response: Thank you for your comments and your thorough evaluation of our manuscript.

In this study, we have conducted only theoretical investigations to explore the methane sensing capabilities of the proposed SnO_x/GO heterostructure. While our results demonstrate promising sensitivity, particularly highlighted by an extinction coefficient of 8.0, we acknowledge that experimental validation is essential for establishing the sensor's practical applicability. We also agree that future experimental validation is essential and plan to pursue this in subsequent studies.

Moreover, there is often a significant gap between theoretical simulation results and actual sensing performance. Theoretical studies, such as those based on Density Functional Theory (DFT), are typically conducted under idealized and highly controlled conditions, assuming perfect crystalline structures, isolated gas molecules, and a vacuum environment. These simulations offer valuable insights into fundamental interactions, such as adsorption energies and charge transfer mechanisms. However, simulations often neglect dynamic effects like adsorption/desorption kinetics, response and recovery times, and long-term material degradation. Even if a material demonstrates strong gas interaction theoretically, practical performance may be hindered by challenges in device fabrication, signal transduction, and reproducibility. Therefore, while theoretical models are essential for guiding material design and predicting potential sensing behavior, experimental validation is crucial to confirm these predictions and assess the material's reliability and efficiency in practical applications.

Author action: We updated the manuscript by modifying/adding some sentences at page 14 and 15 of the revised manuscript.

Reviewer#3, Comment # 3: The conclusion states that the SnOx/GO heterostructure holds potential in optoelectronics and gas sensing, but does not discuss the limitations for practical applications. For instance, does the high absorption coefficient of Type-C also entail issues of thermal stability or durability? Are there process challenges in scaling up the fabrication of SnOx/GO heterostructures? Moreover, has the long-term stability or environmental adaptability of methane sensing been evaluated?

Author response: Thank you for your comments and your thorough evaluation of our manuscript.

We appreciate your recognition for acknowledging the potential of the SnO_x/GO heterostructure in optoelectronics and gas sensing applications. However, we agree that it is essential to address the limitations that may affect its practical implementation. There is possibility that the high absorption coefficient indeed raises concerns regarding thermal stability and durability[27]. High absorption may lead to an increase in the local temperature during operation, which could negatively impact the material's stability, especially at elevated temperatures. Thermal degradation or material fatigue over prolonged use is a critical factor that must be thoroughly evaluated to ensure the material maintains its performance over time, particularly in environments with fluctuating temperatures [28].

Additionally, while scalability is a key aspect for the practical application of any novel material, fabrication challenges for SnO_x/GO heterostructures could arise during the scaling-up process. Achieving consistent quality and uniformity of the heterojunction in large-scale production may require careful optimization of synthesis methods and conditions. Material processing techniques, such as controlled deposition or layer-by-layer assembly, need to be evaluated for their ability to produce SnO_x/GO heterostructures on a larger scale without compromising performance [29].

Furthermore, while the SnO_x/GO heterostructure shows significant potential for applications in optoelectronics and gas sensing, aspects such as its thermal stability, scalability, and long-term performance under real-world conditions remain to be explored experimentally. These investigations are beyond the scope of the present theoretical study and can be addressed in future work.

Author action: We updated the manuscript by modifying/adding some sentences at page 10 of the revised manuscript.

Reviewer#3, Comment # 4: The heterostructures are categorized into four types (Type-A to Type-D) based on the oxygen molar fraction of SnOx. Why is oxygen content chosen as the classification criterion? How does this classification influence the differences in optoelectronic properties of the materials?

Author response: Thank you for your comments and your thorough evaluation of our manuscript.

We performed Density Functional Theory (DFT) simulations to model SnO_x molecules with varying oxygen content. The non-stoichiometry of SnO_x was systematically adjusted by modifying the relative proportions of SnO and SnO_2. For each compositional ratio, we calculated the corresponding value of (x), representing the oxygen mole fraction or the average number of oxygen atoms per Sn atom. In this study, we have considered four different ratios of SnO_2 and SnO to systematically vary the oxygen content in SnO_x, resulting in compositions with (x)=1.5, (x)=1.33, (x)=1.25, and (x)=1.20. These non-stoichiometric compositions represent intermediate states between pure SnO_2 and SnO are categorized as follows: $SnO_{1.5}$ (Type A, oxygen-rich), $SnO_{1.33}$ (Type B), $SnO_{1.25}$ (Type C), and $SnO_{1.20}$ (Type D, oxygen-deficient).

The $SnO_{1.5}$ corresponds to a mixture of 1 mole of SnO and 1 mole of SnO_2, yielding an average oxygen-to-tin ratio (x)=1.5. The value of oxygen mole fraction (x) is calculated using the formula:

(x)=Number of O atoms in the composition/Number of Sn atoms in the composition

In this case, the total O atoms = 1 (from SnO) + 2 (from SnO_2) = 3 and the total Sn atoms = 1 (from SnO) + 1 (from SnO_2) = 2, yielding (x)=3/2=1.5.

This approach allows precise control over the oxygen content in SnO_x, facilitating the study of how varying non-stoichiometry influences the material's optoelectronic properties. Similarly, a composition of $SnO_{1.33}$ corresponds to a mixture of 1 mole of SnO_2 and 2 moles of SnO, yielding an average oxygen-to-tin ratio (x)=1.33. For $SnO_{1.25}$, the mixture consists of 1 mole of SnO_2 and 3 moles of SnO, resulting in (x)=1.25. Likewise, $SnO_{1.20}$ represents a composition with 1 mole of SnO_2 and 4 moles of SnO, giving an average value of (x)=1.20. Each of these compositions reflects a different ratio of SnO_2 to SnO and is used to model non-stoichiometric SnO_x phases by varying the oxygen content systematically.

The phenomenon of increased reflectivity and decreased photo absorption caused by methane adsorption on the surface of graphene oxide can be primarily attributed to changes in charge density, density of states (DOS), and the dielectric constant. When methane molecules adsorb onto the graphene oxide surface, they induce a redistribution of electronic charge at the interface, altering the local charge density. This redistribution modifies the electronic environment, leading to subtle shifts in the DOS, particularly near the Fermi level, which affects the probability of electronic transitions under incident light [23]. As shown in the Density of states (DOS) plots in Figure 5, an increase in methane concentration (from Type-I to Type-IV) results in significant modifications in the electronic structure of the SnO_x/GO heterostructure. Specifically, with higher CH_4 adsorption, the density of states near the Fermi level becomes more pronounced, indicating stronger electronic interactions between methane molecules and the sensing surface. These changes can be attributed to enhanced

charge transfer between methane and the SnO_x/GO system. At low concentrations, methane introduces only slight perturbations to the DOS, mostly due to weak physisorption. However, as concentration increases, stronger chemisorption occurs, leading to additional localized states within the bandgap and near the Fermi level. This results in a shift in the Fermi level results in an increased in charge redistribution and electronic coupling between adsorbed methane and surface atoms. These DOS changes directly impact the material's photoabsorption and sensing behavior. The introduction of new states modifies the allowed optical transitions, increasing the absorption of visible and UV light. These findings are consistent with reported trends in literature, where increased DOS near the Fermi level upon gas adsorption correlates with improved sensing properties [24], [25]. Furthermore, these electronic changes influence the material's complex dielectric constant—specifically, the real part (ε_1)which governs reflectivity, and the imaginary part (ε_2), which determines absorption. Methane adsorption tends to reduce ε_2 resulting in lower optical absorption, while changes in ε_1 can enhance reflectivity [26].

Author action: We updated the manuscript by modifying/adding some sentences in the abstract at page 4, 9 and 10 of the revised manuscript.

Reviewer#3, Comment # 5: Experimental results show that the Type-C heterostructure exhibits the highest absorption coefficient (~1.8×10^5 cm^{-1}) near 100 nm, while Type-II shows the highest extinction coefficient (~8.0) near 1000 nm. Can these properties be synergistically optimized by tuning the oxygen content or methane adsorption level? Is it possible to balance both characteristics?

Author response: Thank you for your comments and your thorough evaluation of our manuscript.

Furthermore, the distinct optical properties exhibited by different SnO_x/GO heterostructure types, specifically, the high absorption coefficient of Type-C (~1.8×10^5 cm^{-1} at ~100 nm) and the high extinction coefficient of Type-II (~8.0 near 1000 nm), highlight the critical role of oxygen content in tuning the material's optoelectronic response. These two features correspond to different spectral regions and mechanisms of interaction: the absorption coefficient signify how strongly the material absorbs photons (useful for UV or high-energy applications), whereas the extinction coefficient encapsulates both absorption and scattering effects, more relevant for visible to near-infrared applications such as photodetection and gas sensing.

The potential to synergistically optimize these properties lies in the tunable composition of SnO_x. As SnO_x is a mixed-phase oxide comprising varying ratios of SnO (narrower bandgap) and SnO_2 (wider bandgap), adjusting the oxygen molar fraction effectively alters the electronic band structure, density of states, and optical transitions. For instance, Type-C SnO_x/GO having SnO-rich compositions (relatively having lower bandgap), tend to show enhanced absorption (~1.8×10^5 cm^{-1} at ~100 nm) in the UV region. This is primarily due to lower energy electronic transitions and stronger localized electron interactions. In contrast, the Type-II SnO_x/GO heterostructure, when exposed to a relatively higher concentration of methane molecules, exhibits significant charge density redistribution and modifications in the density of states. These changes enhance charge separation and dielectric behavior, resulting in a pronounced increase in extinction in the near-infrared (NIR) region. Additionally, methane adsorption plays a significant role in altering the charge distribution, local electronic states, and dielectric properties at the interface of the heterostructure. According to our DFT simulations, the adsorption of methane leads to shifts in the Fermi level, changes in the local charge density, and slight modifications in the optical response due to perturbations in the electronic structure. This suggests that by controlling the exposure to methane, the optical behavior of the material could be dynamically tuned, offering a secondary mechanism for adjusting its properties in response to varying environmental conditions.

Author action: We updated the manuscript by modifying/adding some sentences at page 10 and 11 of the revised manuscript.

Reviewer#3, Comment # 6: The study notes that methane adsorption significantly alters the refractive index and extinction coefficient of the heterostructures, yet stability tests under actual gas environments are not addressed. In practical applications, would prolonged exposure to methane cause drift in the material's optoelectronic properties due to chemical degradation or structural reconstruction?

Author response: Thank you for your comments and your thorough evaluation of our manuscript.

This theoretical study reveals that methane adsorption induces notable changes in the refractive index and extinction coefficient of the SnO_x/GO heterostructures indicating strong potential for methane sensing. Moreover, there is often a significant gap between theoretical simulation results and actual sensing performance. Theoretical studies, such as those based on Density Functional Theory (DFT), are typically conducted under idealized and highly controlled conditions, assuming perfect crystalline structures, isolated gas molecules, and a vacuum environment. These simulations offer valuable insights into fundamental interactions, such as adsorption energies and charge transfer mechanisms. However, simulations often neglect dynamic effects like adsorption/desorption kinetics, response and recovery times, and long-term material degradation. Even if a material demonstrates strong gas interaction theoretically, practical performance may be hindered by challenges in device fabrication, signal transduction, and reproducibility. Therefore, while theoretical models are essential for guiding material design and predicting potential sensing behavior, experimental validation is crucial to confirm these predictions and assess the material's reliability and efficiency in practical applications.

Furthermore, while the SnO_x/GO heterostructure shows significant potential for applications in optoelectronics and gas sensing aspects such as its thermal stability, scalability, and long-term performance under real-world conditions remain to be explored experimentally. These investigations are beyond the scope of the present theoretical study and can be addressed in future work.

Author action: We updated the manuscript by modifying/adding some sentences at page 14 and 15 of the revised manuscript.

Reviewer#3, Comment # 7: Compared to pure SnO_2 or graphene-based sensors, what are the main advantages of the SnOx/GO heterostructure? The paper mentions "low operating temperature"—what is the specific value? Has its performance been experimentally compared with that of existing commercial sensors?

Author response: Thank you for your comments and your thorough evaluation of our manuscript.

In comparison to pure SnO_2 or graphene-based sensors, the SnO_x/GO heterostructure offers several distinct advantages. The integration of SnO_2 with graphene oxide (GO) significantly enhances charge transfer and surface interactions, improving the overall sensitivity and selectivity of the sensor [2]. The heterostructure benefits from the high surface area of GO, which facilitates better adsorption of target molecules, while the semiconducting properties of SnO_2 provide a robust platform for gas detection. Additionally, the hybrid structure offers better stability, improved conductivity, and increased photocatalytic activity, which are advantageous for applications in methane sensing [8]. These combined properties make the SnO_x/GO heterostructure more more efficient in methane detection, compared to pure SnO_2 or graphene based sensors, which may not exhibit the same level of sensitivity and stability. The SnO_x, a well-known metal oxide, is renowned for its tunable bandgap and high stability, making it ideal for gas sensing and optoelectronic applications [9]. GO, on the other hand, is a two-dimensional material with remarkable surface area and tunable electronic properties [10], which enhances the performance of the composite when integrated with SnO_x. The combination of the optical and electronic properties of graphene oxide (GO) with the semiconducting

nature of SnO_x-based composites establishes a robust and versatile platform for the development of optoelectronic devices.

Regarding the operating temperature, the "low operating temperature" mentioned in the manuscript typically refers to ambient or room temperature conditions, which ranges around 25°C - 250 °C [6]. However, this value can vary depending on the specific application and the sensitivity required for the sensor.

Since our study is based on computational simulations, we have not yet performed any experimental comparisons with existing commercial sensors. However, we acknowledge that experimental validation, including performance comparison with commercial sensors, is critical for assessing the practical applicability of the SnO_x/GO heterostructure in methane detection. These investigations are beyond the scope of the present theoretical study and can be addressed in future work.

Author action: We updated the manuscript by modifying/adding some sentences at page 2 of the revised manuscript.
